# Patient- and endoscopist-related risk factors and etiological categorization of post-colonoscopy colorectal cancer

**DOI:** 10.1055/a-2566-3380

**Published:** 2025-05-16

**Authors:** Esly Lemmen, Judith Sluiter-Post, Karlijn van Stralen, Ellert van Soest

**Affiliations:** 1 1190 Faculty of Medicine, Vrije Universiteit Amsterdam, Amsterdam, Netherlands; 2 3670 Department of Gastroenterology, Spaarne Gasthuis, Hoofddorp, Netherlands; 3 3670 Spaarne Gasthuis Academy, Spaarne Gasthuis, Hoofddorp, Netherlands

**Keywords:** Endoscopy Lower GI Tract, Polyps / adenomas / ..., Colorectal cancer, Quality and logistical aspects, Quality management, Performance and complications, CRC screening

## Abstract

**Background and study aims:**

Colonoscopy is considered to be the gold standard for detecting colorectal cancer. However, this technique is not flawless and post-colonoscopy colorectal cancers (PCCRCs) can occur. Therefore, we investigated the association between patient- and endoscopist-related risk factors and occurrence of PCCRC.

**Patients and methods:**

A matched case-control study design was employed. Data from the national colorectal cancer screening program, along with medical records, were used to identify patients diagnosed with colorectal cancer from 2012 until 2022 who had a negative colonoscopy in the 4 years preceding the diagnosis. Patients with colorectal cancer (cases) were matched in a 1:2 ratio with patients without colorectal cancer (controls) based on the date of the negative index colonoscopy of the cases. Analyses at the patient and endoscopist level were conducted to assess factors associated with PCCRC occurrence. Root cause analysis, using the World Endoscopy Organization categorization, was performed to identify possible PCCRC causes.

**Results:**

Of 72,975 colonoscopies, 61 PCCRC cases (62% male, mean age 77 years) were found, resulting in an incidence of 22 per 100,000 patient years. Root cause analysis showed that over 75% of PCCRCs could be classified as a possibly missed lesion during index colonoscopy. Endoscopists with a higher mean number of adenomas per colonoscopy had significantly lower PCCRC incidence.

**Conclusions:**

Endoscopists detecting more adenomas had a substantially lower PCCRC incidence in their patients. Therefore, endoscopist performance is a crucial marker of PCCRC and may serve as a quality control measure for colonoscopy.

## Introduction

Colorectal cancer (CRC) is a significant global health issue, ranking as the third most common malignancy worldwide 1 2 3 4 . Moreover, it is among the most fatal forms of cancer, with incidence rates continuing to rise 1 2 3 4 . In 2020, nearly 2 million new cases were diagnosed 2 . Colonoscopy is considered the gold standard for detection of CRC and removal of adenomas and polyps 5 6 . More importantly, utilization of colonoscopy for detecting and removing these (pre)cancerous lesions has been associated with reductions in both the incidence and mortality of CRC 7 8 .

Despite its status as the gold standard, this technique is not infallible 5 6 . Post-colonoscopy CRC (PCCRC) can still occur 5 6 9 10 . This term refers to CRC diagnosed after a colonoscopy in which initially no cancer was found 9 . According to the World Endoscopy Organization (WEO), CRCs that occur within 4 years after a colonoscopy are unlikely to be new cancers. Previous studies have reported PCCRC incidences ranging from 2% up to 10% 5 6 11 12 13 14 .

Known patient-related risk factors for PCCRC include right-sided tumors, older age, female sex, hereditary CRC syndromes and presence of large polyps during index colonoscopy 15 16 17 . It has been suggested that the majority of PCCRC cases are preventable 18 . Furthermore, a low adenoma detection rate (ADR) by endoscopists has been identified as an endoscopist-related risk factor for PCCRC 11 19 . Consequently, PCCRC rates can serve as a quality indicator for a hospital or endoscopist. However, the association between mean number of adenomas per colonoscopy (MAP) and mean number of adenomas per positive colonoscopy (MAP+) and PCCRC remains less clear 20 21 22 . In addition to general risk factors, understanding the etiology behind the PCCRCs is crucial for potential prevention in the future 10 .

Therefore, the primary aim of this study was to assess the association between the occurrence of PCCRC and risk factors on multiple levels; both patient- and endoscopist-related. The secondary aim was to classify PCCRC cases into etiological categories according to the WEO classification system 9 .

## Patients and methods

### Study setting and design

A matched case-control study design was used to examine the association between patient- and endoscopist-related risk factors and PCCRC. All PCCRC cases diagnosed in a teaching hospital in the Netherlands between January 2012 and October 2022 were identified. This teaching hospital serves an adherence area of approximately 500,000 people. Hospital care is organized regionally with affiliated general practices. When patients undergo colonoscopy in our hospital, they virtually always return for a follow-up colonoscopy, except when a patient has moved. PCCRC cases were defined as follows: 1) diagnoses of colorectal cancer through colonoscopy; and 2) CRC diagnosed between 6 months and 48 months after an initial negative colonoscopy (index colonoscopy). As defined by the WEO, a cut-off point at 4 years was used to identify cases that were unlikely to be new CRCs 9 . A negative colonoscopy referred to a colonoscopy in which no CRC was detected. After establishing the PCCRC group, a control group was formed from the same pool of patients, all of whom were under the care of an endoscopist and had an indication for colonoscopy, by matching patients without colorectal cancer in a 1:2 ratio based on the index colonoscopy date. This study was reviewed and approved by the Spaarne Gasthuis institutional review board (reference number 2022.0079).

### Data collection

Patient characteristics and colonoscopy procedure data were obtained from hospital-based medical records. A national digital automated archive (PALGA) that collects pathological-anatomical records was used to extract the number of colorectal cancer patients from 2012 until October of 2022 and to pair PCCRC patients to their pathological tumor characteristics 23 . Data on endoscopists as well as the annual colonoscopy volume and the incidence of PCCRC per 1000 patient-years were also collected during this time period. Endoscopists were gastroenterologists, gastroenterologists in training or surgeons. Among the endoscopists, ten were registered endoscopists in the Dutch national screening program (Bevolkingsonderzoek [BVO]). These screening endoscopists upheld a prospective registry of quality indicators (ADR, percentage of screening colonoscopies that detect at least one adenoma, as well as MAP and MAP+), and have completed an e-learning module and practical testing of colonoscopy and polypectomy skills 24 . All endoscopists were included in the patient-level analysis, but only the 10 screening endoscopists were included in incidence and endoscopist-level analysis.

### Root cause analysis of PCCRC

Root cause analysis of PCCRC was conducted using the WEO categorization approach, classifying each case under one of five most probable explanations for PCCRC 9 . Two researchers performed the WEO classification independently, with differences resolved through discussion. Index colonoscopy was considered complete and adequate if there was documented adequate bowel preparation and evidence of cecal intubation.

### Statistical analysis

We performed various analyses, each on different “levels” to assess patient- and endoscopist-level factors associated with occurrence of PCCRC. The analysis was structured as follows.

#### Patient level

At the patient-level analysis, logistic regression was used to compare baseline characteristics of cases and controls. Associations between occurrence of PCCRC and patient-related factors were assessed using univariate logistic regression models. To account for potential confounding, an additional conditional logistic regression analysis was conducted, adjusting for patient sex, age, Lynch syndrome, tobacco use (ever or current combined) and having one or more adenomas present on index colonoscopy. Odds ratios (ORs), adjusted odds ratios (aORs) and their 95% confidence intervals (CIs) were used to quantify these associations.

To adjust for endoscopist, we used multilevel analyses using ordinal logistic regression. This model incorporated sex, Lynch syndrome, tobacco use (ever or current combined) and presence of one or more adenomas during index colonoscopy as categorical predictors and age, ADR, MAP, and MAP+ as continuous covariates. To account for clustering at the endoscopist level, a repeated-measure structure was applied, with the endoscopist defined as the clustering variable. Cases with missing values in categorical variables were excluded from the analysis. Results were presented as adjusted effect estimates with 95% CIs.

#### Endoscopist level

At the endoscopist level, incidence of PCCRC per 1000 patient-years was calculated for each screening endoscopist. To investigate the association between ADR, MAP, and MAP+ and PCCRC incidence rates, a linear regression analysis was performed. Regression coefficients with 95% CIs were reported.

All data were analyzed using IBM SPSS Statistics Version 27.0 (IBM Corp., Armonk, New York, United States). *P* ≤0.05 was considered statistically significant.

## Results

### Subject characteristics

During the 10-year period, 72,975 colonoscopies were performed, resulting in diagnosis of CRC in 3,404 patients. Sixty-one patients were identified as PCCRC (cases) and were matched to 122 controls ( Table 1 ). Six of 61 cases underwent screening colonoscopies as part of the Dutch national screening program, all of which followed a positive fecal immunochemical test (FIT). In addition, two other cases were referred by their general practitioner after a positive FIT result. Other indications were surveillance after adenoma/CRC, rectal bleeding, anemia, change in defecation pattern, abdominal complaints, familial risk of CRC or Lynch syndrome, and inflammatory bowel disease. In both the case and control group, the most common indications were surveillance, rectal bleeding, changes in defecation patterns, and participation in the screening program.

**Table:** **Table 1** Baseline characteristics of study population and association with PCCRC.

Characteristics	Cases (n = 61)	Controls (n = 122)	Total (n = 183)	OR	95% CI	*P* value
Sex (n (%))						
Male	38 (62.3)	47 (38.5)	85 (46.4)	2.64	1.40–4.97	0.003
Female	23 (37.7)	75 (61.5)	98 (53.6)			
Age (mean ± SD)	77.1 ± 8.0	67.9 ± 16.0	71.0 ± 14.5	1.05	1.03–1.08	< 0.001
Lynch syndrome (n (%))	4 (6.6)	1 (0.8)	5 (2.7)	8.49	0.93–77.70	0.058
Tobacco use (ever used) (n (%))	47 (77.0)	66 (54.1)	113 (61.7)	2.41	1.17–4.97	0.017
Adenoma(s) present in index colonoscopy (n (%))	29 (47.5)	24 (19.7)	53 (29.0)	3.70	1.89–7.25	< 0.001
Quality bowel preparation (BBPS) (n (%))				0.46	0.17–1.27	0.13
Adequate ≥ 6	41 (67.2)	101 (82.8)	142 (77.6)			
Not adequate ≤5	8 (13.1)	9 (7.4)	17 (9.3)			
Missing	12 (19.7)	12 (9.8)	24 (13.1)			
Endoscopist specialty (n (%))						
Gastroenterologist	50 (82.0)	107 (87.7)	157 (85.8)	Ref		
Gastroenterology resident	9 (14.7)	13 (10.7)	22 (12.0)	1.48	0.59–3.70	0.40
Surgeon	2 (3.3)	1 (0.8)	3 (1.6)	4.28	0.38–48.32	0.24
Missing		1 (0.8)	1 (0.6)			
Screening endoscopist, performing screening colonoscopies (BVO) (n(%))	45 (73.8)	98 (80.3)	143 (78.1)	0.69	0.33–1.42	0.31
BBPS, Boston Bowel Preparation Scale; BVO, Bevolkingsonderzoek (Dutch national screening program); CI, confidence interval; OR, odds ratio; PCCRC, post-colonoscopy colorectal cancer; SD, standard deviation.						

### Patient-related risk factors

Mean age of PCCRC cases was 77.1 years (± SD 8.0) compared with 67.9 years (±SD 16.0) for controls ( *P* <0.001). Of the cases 62.3% (38/61) were male, versus 38.5% (47/122) of the controls ( *P* = 0.003). Significant differences were observed between cases and controls in terms of current or ever tobacco use (77.0% versus 54.1%, *P* = 0.017) and presence of adenomas during index colonoscopy (47.5% versus 19.7%, *P* < 0.001). Lynch syndrome was diagnosed in 6.6% of cases (4/61) and 0.8% of controls (1/122) ( *P* = 0.058). Patients with adenomas at index colonoscopy had a 3.70-fold increased risk (95% CI 1.89–7.25) of PCCRC compared with those without adenomas. No significant difference was found between the groups in terms of bowel preparation quality (adequate quality defined as ≥6, inadequate as ≤5; *P* = 0.13). Key characteristics of cases and controls are detailed in Table 1 .

Patient-level analysis showed significant inverse associations for MAP (aOR 0.06; 95% CI 0.01–0.47; *P* = 0.007), MAP+ (aOR 0.12; 95% CI 0.02–0.62; *P* = 0.011), and ADR (aOR 0.87; 95% CI 0.78–0.97; *P* = 0.009) after adjusting for sex, age, presence of one or more adenomas during index colonoscopy, smoking, and Lynch syndrome ( Table 2 ). The results indicate that as MAP, MAP+, and ADR increased, odds of developing PCCRC significantly decreased, underscoring potential protective effects of these factors in relation to PCCRC.

**Table:** **Table 2** ADR, MAP, and MAP+ and risk of PCCRC.

Characteristics	aOR	95% CI	*P* value
ADR	0.87	0.78–0.97	0.009
MAP	0.06	0.01–0.47	0.007
MAP+	0.12	0.02–0.62	0.011
aOR, adjusted odds ratio; ADR, adenoma detection rate; CI, confidence interval; MAP, mean number of adenomas per colonoscopy; MAP+, mean number of adenomas per positive colonoscopy; PCCRC, post-colonoscopy colorectal cancer.			

After adjustment for endoscopist, using multilevel analyses, similar factors were identified. A higher MAP for the endoscopist was associated with lower PCCRC occurrence (B = -2.57; 95% CI -4.28 to -0.86; *P* = 0.003). Individuals with adenomas had significantly higher risk of PCCRC (B = 1.22; 95% CI 0.17–2.28; *P* = 0.023). Older age was associated with an increased risk of PCCRC (B = 0.06; 95% CI 0.01–0.10; *P* = 0.011). Presence of Lynch syndrome was linked to higher risk of PCCRC (B = 2.64; 95% CI; 0.95–4.33; *P* = 0.002) ( Table 3 ).

**Table:** **Table 3** Combined analyses including both patient and endoscopist factors, using adjustment for endoscopist.

Characteristics	B (Adjusted)	95% CI	*P* value
Sex, male	0.41	–0.72 to 1.54	0.48
Age	0.06	0.01 to 0.10	0.011
Lynch syndrome	2.64	0.95 to 4.33	0.002
Tobacco use (ever used)	0.87	–0.16 to 1.91	0.097
Adenoma(s) present in index colonoscopy	1.22	0.17 to 2.28	0.023
ADR	0.03	–0.02 to 0.08	0.21
MAP	–2.57	–4.28 to -0.86	0.003
MAP+	0.20	–0.83 to 1.23	0.70
ADR, adenoma detection rate; B (Adjusted), adjusted regression coefficient; CI, confidence interval; MAP, mean number of adenomas per colonoscopy; MAP+, mean number of adenomas per positive colonoscopy.			

### Endoscopist-related risk factors

Most index colonoscopies (78.1%) were performed by screening endoscopists. Sixteen of 61 cases and 24 of 122 controls had their index colonoscopy performed by an endoscopist not part of the national screening program, although this difference was not statistically significant (OR 0.69; 95% CI 0.33–1.42; *P* = 0.31). More colonoscopies of cases were performed by surgeons ( *P* = 0.24) or residents ( *P* = 0.40) than controls, but these differences were also not statistically significant ( Table 1 ).

Forty-five cases and 98 controls had their index colonoscopy performed by screening endoscopists. Screening endoscopist performance characteristics are presented in Table 4 . Among the 10 screening endoscopists, ADR ranged from 44.3% to 67.2%, MAP from 0.99 to 2.08, and MAP+ from 2.10 to 3.42. Higher MAP was associated with significantly lower incidence of PCCRC; each point increase in MAP reduced incidence by 0.17 per 1000 patient-years (95% CI –0.34 to –0.007, *P* = 0.043). Similar, although not statistically significant, results were observed for MAP+ (–0.11; 95% CI –0.28 to 0.06, *P* = 0.16) and ADR (–0.006; 95% CI -0.017 to 0.004; *P* = 0.18). No effect was found for number of colonoscopies performed (0.011 per 1000 colonoscopies, 95% CI –0.06 to 0.08, *P* = 0.72).

**Table:** **Table 4** Characteristics per screening endoscopist.

Screening endoscopists	#PCCRCS per 1000 patient-years	ADR	MAP	MAP+	# Colonoscopies 2012–2022	#Patient- years 2012–2022
1	0.37	44.3%	0.99	2.12	7503	21859
2	0.37	60.4%	1.21	2.35	5761	16163
3	0,29	53.9%	1.17	2.46	4983	13809
4	0.28	58.6%	1.08	2.10	4106	10791
5	0.23	61.5%	1.77	3.17	3133	8818
6	0.22	63.7%	1.73	2.71	4882	13750
7	0.16	60.5%	1.28	2.35	6506	18374
8	0.12	47.8%	1.14	2.15	5663	16383
9	0.11	66.1%	2.08	3.42	6128	17939
10	0.07	67.2%	1.93	2.82	4785	14450
Median	**0.22**	**60.4%**	**1.21**	**2.35**		
Total					**53450**	**152336**
ADR, adenoma detection rate; MAP, mean number of adenomas per colonoscopy; MAP+, mean number of adenomas per positive colonoscopy; PCCRC, post-colonoscopy colorectal cancer.						

### Etiology of PCCRCs according to WEO classification system

Among the 61 diagnosed PCCRC cases, underlying etiology could be classified in 88.5% (54/61). Of these, 31 cases (57.4%) were classified as possibly missed despite adequate index colonoscopic examination, meaning the lesion was present but not detected at during the procedure despite appropriate technique and bowel preparation. In contrast, 10 cases (18.5%) were missed without adequate examination, where suboptimal bowel preparation or incomplete visualization of the colon may have contributed to failure to detect the lesion. Together, these account for almost 76% of lesions that were undetected during index colonoscopy.

In addition, one lesion (1.9%) was detected but not resected at time of colonoscopy, and 10 lesions (18.5%) were likely resected incompletely, meaning the residual lesion remained after attempted removal. Of the patients with a possible missed lesion and inadequate examination, seven had incomplete colonoscopies due to poor bowel preparation. The majority of these patients were scheduled for repeat colonoscopies within 3 to 5 years. In two cases, cecal intubation was not achieved, necessitating follow-up with computed tomography-colonography. In one case, only a portion of the colon was adequately examined, resulting in an observational approach without further follow-up.

**Fig. 1:**
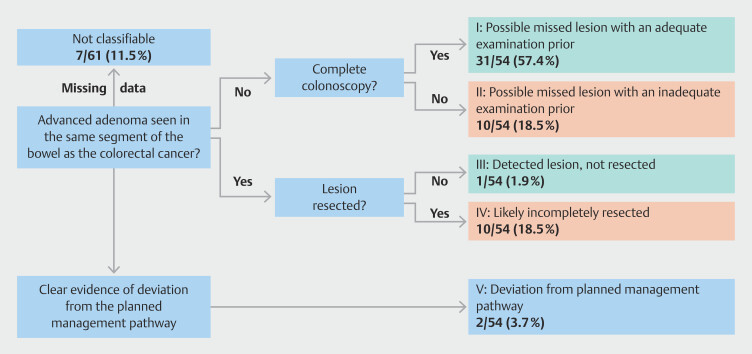
Diagram of the most plausible explanation for PCCRC based on WEO root cause analysis.

Two patients (3.7%) deviated from the recommended management pathway due to either non-compliance or refusal of follow-up colonoscopy. Fig. 1 gives a visual display of this distribution.

## Discussion

We showed that endoscopist performance has a major impact on occurrence of PCCRC. Approximately three-quarters of PCCRC cases could be explained by a possible missed lesion, consistent with previous studies that have used the WEO categorization system 10 18 25 26 . Incomplete resection accounted for 18.5% of PCCRCs, aligning with findings in the literature ranging from 7.0% to 19.0% 10 18 25 . It is important to note that this approach to categorizing PCCRC cases based on most plausible etiology has limitations, including lack of validation, considering the complexities of cancer biology and sojourn time.

The results of our study reveal notable variability among the 10 screening endoscopists regarding ADR, MAP and MAP+. This variability is crucial because it highlights differences in endoscopist performance and potentially the impact on patient outcomes. Even with this small number of endoscopists, we found an inverse association between MAP and occurrence of PCCRC at both patient and endoscopist levels. This finding suggests that patients whose colonoscopies are performed by endoscopists with a lower MAP may be at higher risk for PCCRC. When both ADR, MAP+, and MAP were included in a single model, only MAP remained, showing the high correlation between the various factors. Previous studies have reported an inverse association between ADR and PCCRCs or interval cancers 11 19 27 . Although ADR is considered one of the most important quality indicators of colonoscopy, Lee et al. suggested that parameters such as MAP and MAP+ might provide added value because they reflect not only the ability of an endoscopist to detect one adenoma, but also the number of adenomas 20 28 29 . To our knowledge, few studies have investigated associations between MAP or MAP+ and PCCRC, of which Anderson et al. also demonstrated that higher MAP reduced risk of PCCRC 24 29 30 . One study also found an inverse association between MAP or MAP+ and interval PCCRC in FIT -positive colonoscopies 19 . Denis et al. even stated that MAP should be considered as the new gold standard to evaluate neoplasia yield 31 . Unfortunately, a uniform benchmark for these parameters is still lacking 31 .

Differences between cases and controls were found in patient factors such as age, sex, smoking, Lynch syndrome, and presence of adenomas during index colonoscopy. In line with previous studies, having Lynch syndrome and older age were associated with increased risk of PCCRC 13 15 17 32 33 . PCCRC cases were more likely to be male, which contrasts with findings from some other studies 32 33 . This study combined current use and ever use of tobacco, which was found to be associated with PCCRC. Smoking is a known risk factor for adenomas, which could explain the observed association 34 . Nearly half of PCCRC cases had one or more adenomas present during index colonoscopy, compared with 19.7% in the control group. Patients with one or more adenomas had over threefold higher odds (95% CI 1.89–7.25) of developing PCCRC than patients without adenomas during index colonoscopy, which remained significant after adjustment for endoscopist. This finding is supported by prior studies with similar outcomes 16 33 .

This study highlights the importance for endoscopists to improve their ADR, MAP+, and particularly MAP, in order to reduce PCCRC cases. A previous study suggested that training, benchmarking, and feedback are the most effective methods to improve ADRs 35 . Further research is needed to establish benchmarks for additional quality parameters and MAP and MAP+. In addition, we advocate for an adequate, preferably automated, quality colonoscopy registry including the PCCRC rate per endoscopist, which could help establish benchmarks for these important quality indicators.

A strength of this study is analyzing associations between PCCRC and ADR, MAP and MAP+ at patient and endoscopist levels. In addition, we used a predefined PCCRC definition and recommended descriptors for the most plausible PCCRC etiology.

Still, this study has some limitations. It was retrospective study, and therefore, relies on the presumption of solid available data. Moreover, all patients are referred within their catchment area of the hospital. However, it is possible that patients moved, or that patients are unhappy with the previous negative diagnosis, and therefore, received their PCCRC diagnosis in another hospital. This will have led to underestimation of incidence of PCCRC. However, we do not believe that this affected the relationship between ADR, MAP, and MAP+ because we believe that it is most likely unrelated to the endoscopist who performed the index colonoscopy.

In addition, cases may have been missed due to lack of unambiguous coding for CRC in patient records, or if patients had their PCCRC diagnosis without a colonoscopy. We did not have the ADR, MAP and MAP+ for all endoscopists because not all were working for the national screening program. However, we did not find a difference between endoscopists who performed and did not perform screening colonoscopies. Despite analyzing over 53,000 colonoscopies, the small number of screening endoscopists could limit endoscopist-level result generalizability. Matching cases and controls by colonoscopy date could also lead to control patient oversampling from the same endoscopists, potentially underestimating results.

## Conclusions

We showed that endoscopist performance is a very important marker for incidence of PCCRC, making it a valuable quality control measure for colonoscopy. Further research is needed to establish benchmarks and investigate the best methods to enhance endoscopist ADR, MAP, and MAP+ in order to reduce these potentially preventable PCCRCs.
